# Association Between hMLH1 Promoter Methylation and Risk of Gastric Cancer: A Meta-Analysis

**DOI:** 10.3389/fphys.2018.00368

**Published:** 2018-04-17

**Authors:** Peng Ye, Yu Shi, Anling Li

**Affiliations:** Department of Laboratory Medicine, Zhongnan Hospital of Wuhan University, Wuhan, China

**Keywords:** gastric cancer, *hMLH1*, methylation, meta-analysis, MSI

## Abstract

**Background:** Human mutL homolog 1 (*hMLH1*) is located on chromosome 3q21-23. As a classic tumor suppressor gene, many researchers have studied the association between *hMLH1* promoter methylation and gastric cancer, but their conclusions were not always consistent. Therefore, we performed a meta-analysis to make a more integrated and precise estimate of the associations.

**Method:** PubMed, EMBASE, and Cochrane Library were retrieved without language restrictions. Data were analyzed by Review Manager 5.2 and Stata 12.0 software. Odds ratio (OR) with 95% confidence interval (95%CI) was used to assess the statistical associations.

**Result:** A total of 39 studies published before January 20, 2018 were included in this study. The results indicated that the frequency of *hMLH1* promoter methylation in gastric cancers was substantially higher than that in non-cancer controls (OR = 7.94, 95%CI = 4.32–14.58, *P* < 0.001). Furthermore, *hMLH1* promoter methylation had considerable associations with lymph node metastasis, microsatellite instability (MSI), and low expression of hMLH1 protein (OR = 1.53, 95%CI = 1.04–2.26, *P* = 0.03; OR = 15.33, 95%CI = 9.26–25.36, *P* < 0.001; OR = 37.86, 95%CI = 18.03–79.50, *P* < 0.001, respectively). No association was found between *hMLH1* promoter methylation and Lauren classification or Helicobacter pylori (HP) infection status.

**Conclusion:** The present study provides evidence that promoter methylation of *hMLH1* is a major causative event in the occurrence and development of human gastric cancer.

## Introduction

Gastric cancer, also known as stomach cancer, continues to be a vital heath threat as the fifth leading cause of cancer and the third leading cause of death from carcinoma globally according to World Health Organization in 2014 (Stewart and Wild, 2014). Owing to inadequate early diagnosis and unclear pathogenesis, gastric cancer was considered to be a high-mortality disease and its 5-year survival rate was reported to be <10% (Orditura et al., 2014). Although the pathogenic mechanisms have not been fully elucidated, some factors including Helicobacter pylori (HP) infection, smoking, and excessive drinking, have been identified to contribute to the tumorigenesis of gastric cancer. In addition, epigenetic silencing of tumor suppressor genes was also thought to play an important part in the genesis of gastric cancer (Tahara and Arisawa, 2015).

DNA methylation is the most common epigenetic mechanism. It is catalyzed by a family of DNA methyltransferases (DNMT) that transfer a methyl group from S-adenyl methionine (SAM) to the fifth carbon of a cytosine residue to form 5- methyl cytosine (5 mC), which is unstable and can spontaneously deaminate to form thymine, thereby affecting gene expression (Moore et al., 2013). Much evidence showed that hypermethylation of normally unmethylated CpG islands in the promoter regions of tumor suppressor genes was strongly related to carcinomas, for instance, *BRCA1* promoter methylation in breast cancer (Zhang and Long, 2015), *GSTP1* promoter methylation in prostate cancer (Jerónimo et al., 2001), *hMLH1* methylation in gastroenteric tumors (Arai et al., 2010). *hMLH1* is one of the human mismatched repair (MMR) genes, which is located on chromosome 3q21-23. As a classic anti-oncogene, the protein encoded by this gene is a component of the DNA mismatch repair pathway which can effectively repair mismatched bases and prevent the accumulation of DNA damage.

During the past decades, the associations between *hMLH1* promoter methylation and the risk or clinicopathological characteristics of stomach cancer have been reported by many researchers. However, the conclusions were not always consistent and some results were unconvincing because of the small sample size. Therefore, we performed a meta-analysis to clarify the role of *hMLH1* gene promoter methylation in the tumorigenesis and development of gastric cancer.

## Methods

### Literature search strategy

PubMed, EMBASE, and Cochrane Library were retrieved to obtain literatures concerning the association between gastric cancer and *hMLH1* promoter methylation without language restrictions. We used the terms as follows: (“*hMLH1*” or “human mutL homolog 1”) and (“methylation”) and (“stomach” or “gastric”) and (“cancer” or “neoplasms” or “carcinoma”). The search results were updated until January 20, 2018. In addition, we also performed manual search for other relevant literatures.

### Selection criteria

The following criteria were used in selecting eligible articles: (a) articles dealing with the association of *hMLH1* promoter methylation with gastric cancers; (b) case-control studies. The major reasons for exclusion of studies: (a) reviews, letters, or case-only articles (b) articles with insufficient data or duplicated data.

### Data extraction and quality assessment

Articles were screened first by reading titles and abstracts, then two reviewers (Y Shi and P Ye) read the entire articles that seemed to fit the inclusion criteria and extracted basic information including the first authors' names, publication years, countries, methylation detection methods, sample sizes, and number of methylation in cases and controls from every eligible study. All included studies concerning *hMLH1* methylation and gastric cancers risk were evaluated by the modified Newcastle-Ottawa scale (NOS) assessment (Jadad et al., 1996) (available at http://www.ohri.ca/programs/clinical_epidemiology/oxford.asp). The latest NOS assessment for case-control studies consists of seven items of methodology which are grouped into three major categories: cases and controls selection, comparability of cases and controls, and ascertainment of exposure. The total score ranges from 0 to 8, and studies with more than four points are considered as qualified. Disagreement was resolved by discussion and consensus.

### Statistical analysis

Data were analyzed by Review Manager 5.2 and Stata 12.0. The strength of the association between *hMLH1* promoter methylation and gastric cancers risk or clinicopathologic characteristics was assessed by pooled OR with corresponding 95% CI. Chi-square test based Q-test and *I*^2^-test were performed to assess heterogeneity among studies. It indicated a lack of heterogeneity if *P* > 0.10 and *I*^2^ < 50%, then the pooled OR would be calculated by using the fixed-effect model in line with the Mantel-Haenszel method. Otherwise, the random-effect model would be used according to the DerSimonian-Laird method. The subgroup analysis was further conducted based on different ethnicities, types of controls, specimen materials, and methods of detecting methylation. The stability of the pooled result was evaluated by sensitivity analysis and potential publication bias was assessed by Begg's test.

## Results

### Study characteristics

**Figure 1:**
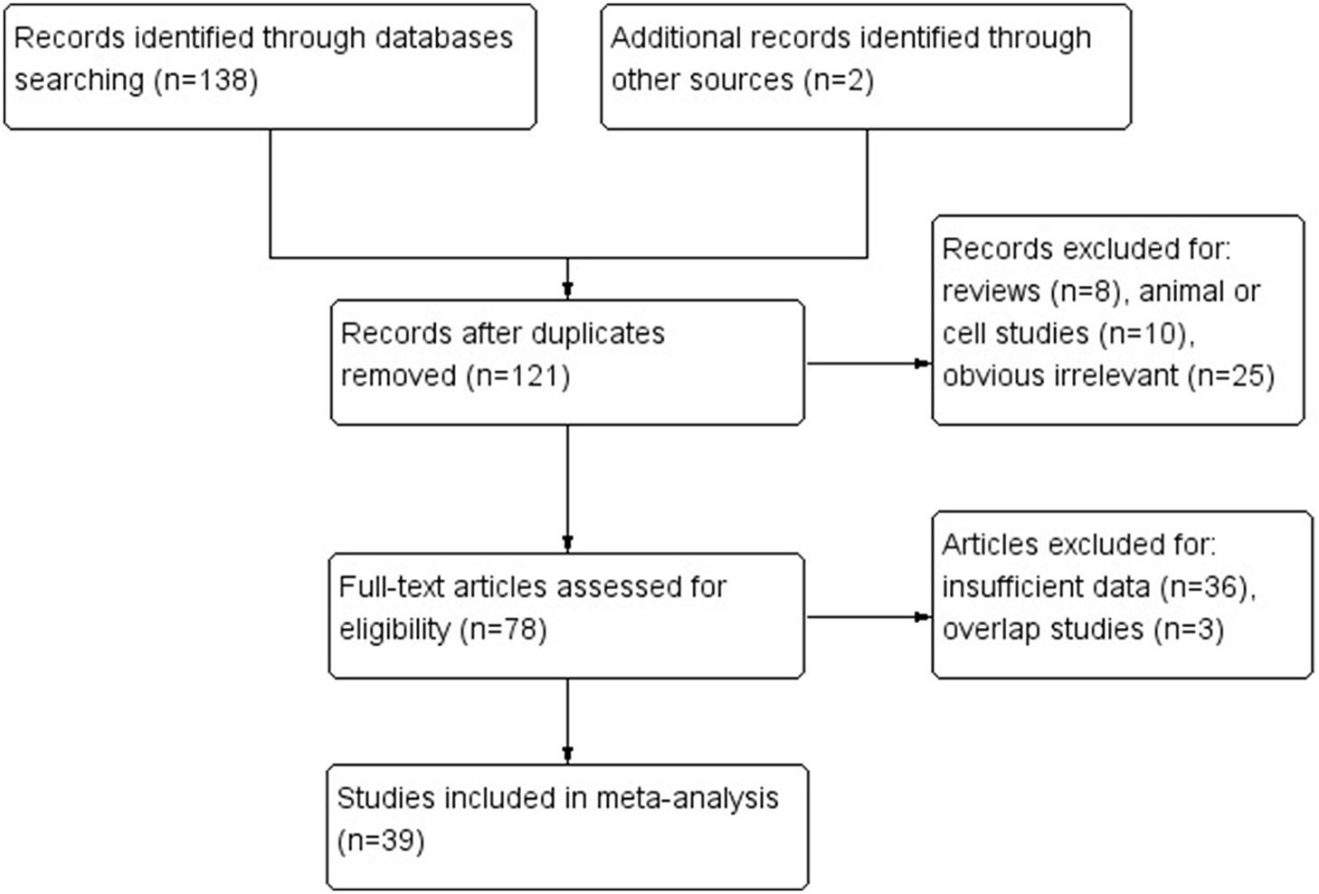
Flow diagram of literature selection.

Based on above selection criteria, 39 studies were included in our research. Twenty-three of them (Suzuki et al., 1999; Bevilacqua and Simpson, 2000; Leung et al., 2001; Oue et al., 2001, 2006; Sakata et al., 2002; Kang et al., 2003; Etoh et al., 2004; An et al., 2005; Hong et al., 2005; Shibata et al., 2006; Kolesnikova et al., 2008; Poplawski et al., 2008; Zhang et al., 2008; Ksiaa et al., 2009; Hiraki et al., 2010; Mikata et al., 2010; Mir et al., 2012; Wani et al., 2012; Song et al., 2013; Xiong et al., 2013; Jin et al., 2014; Liu and Yang, 2015) evaluated the association between hMLIH1 promoter methylation and gastric cancer risk, including 2,182 cases and 2,319 controls; 27 of them (Fleisher et al., 1999; Leung et al., 1999; Suzuki et al., 1999; Toyota et al., 1999; Pinto et al., 2000; Nakajima et al., 2001; Oue et al., 2001, 2003; Sakata et al., 2002; Fang et al., 2003; Sugai et al., 2004; Wu et al., 2004; An et al., 2005; Hong et al., 2005; Kim et al., 2005, 2010; Nan et al., 2005; Kolesnikova et al., 2008; Ferrasi et al., 2010; Hiraki et al., 2010; Mikata et al., 2010; Alves et al., 2011; Song et al., 2013; Xiong et al., 2013; Jin et al., 2014; Moghbeli et al., 2014; Kawanaka et al., 2016) with 2,713 patients investigated the associations of hMLIH1 methylation with clinicopathological features including lymph node metastasis, Lauren's histological type, microsatellite status, Helicobacter pylori (HP) infection status, and hMLH1 protein expression in gastric cancer patients. The flow chart (Figure 1) summarized the study screening process. The main characteristics of these included studies were listed in Table 1.

**Table 1:** Characteristics of studies concerning hMLH1 methylation and gastric cancer risk.

**Author**	**Year**	**Country**	**Case**	**Control**	**Method**	**Materials**	**Control type**	**NOS score**
			**M+/M−**	**M+/M−**				
Liu, L	2015	China	24/26	1/29	MSP	Blood	H	7
Jin, J	2014	China	16/267	0/283	MSP	Tissue	A	7
Song, B	2013	China	17/305	0/313	MSP	Tissue	A	7
Xiong, H. L	2013	China	19/394	0/413	MSP	Tissue	A	6
Wani, M	2012	India	51/19	14/56	MSP	Tissue	A	7
Mir, M. R	2012	India	104/26	82/48	MSP	Tissue	A	7
Mikata, R	2010	Japan	2/19	1/20	MSP	Tissue	A	7
Hiraki, M	2010	Japan	32/17	21/28	Q-MSP	Tissue	A	7
Ksiaa, F	2009	Tunisia	6/62	0/53	MSP	Tissue	A	7
Kolesnikova, E. V	2008	Russia	5/15	2/20	MSP	Blood	H	5
Zhang, K. L	2008	China	25/22	3/28	MSP	Tissue	H	7
Poplawski, T	2008	Poland	6/21	0/25	MSRE-MSP	T/B	H	7
Shibata, D	2006	USA	21/27	0/48	Q-MSP	Tissue	A	6
Oue, N	2006	Japan	8/67	0/10	MSP	Tissue	H	5
Hong, S. H	2005	Korea	26/74	2/236	MSP	T/B	H	4
An, C	2005	USA	14/69	0/82	MSP	Tissue	A	7
Etoh, T	2004	Japan	18/87	15/90	MSP	Tissue	A	7
Kang, G. H	2003	Korea	16/64	0/210	MSP	Tissue	H	4
Oue, N	2001	Japan	11/39	4/46	MSP	Tissue	A	6
Sakata, K	2002	Japan	6/6	5/3	MSP	Tissue	A	6
Leung, W. K	2001	China	9/17	0/25	MSP	Tissue	A	6
Bevilacqua, R. A	2000	Brazil	8/34	0/42	MSRE-MSP	Tissue	A	7
Suzuki, H	1999	Japan	5/56	0/61	COBRA	Tissue	A	6

*M+, methylated; M−, unmethylated; H, heterogeneous; A, autologous; MSP, methylation-specific PCR; QMSP, methylation-specific quantitative PCR; MSRE-MSP, methylation specific restriction enzyme PCR; COBRA, combined bisulfite restriction analysis; T/B, cancer samples were gastric mucosa tissues from gastric cancer patients while control samples were peripheral blood from non-cancer people*.

### Meta-analysis results

**Figure 2:**
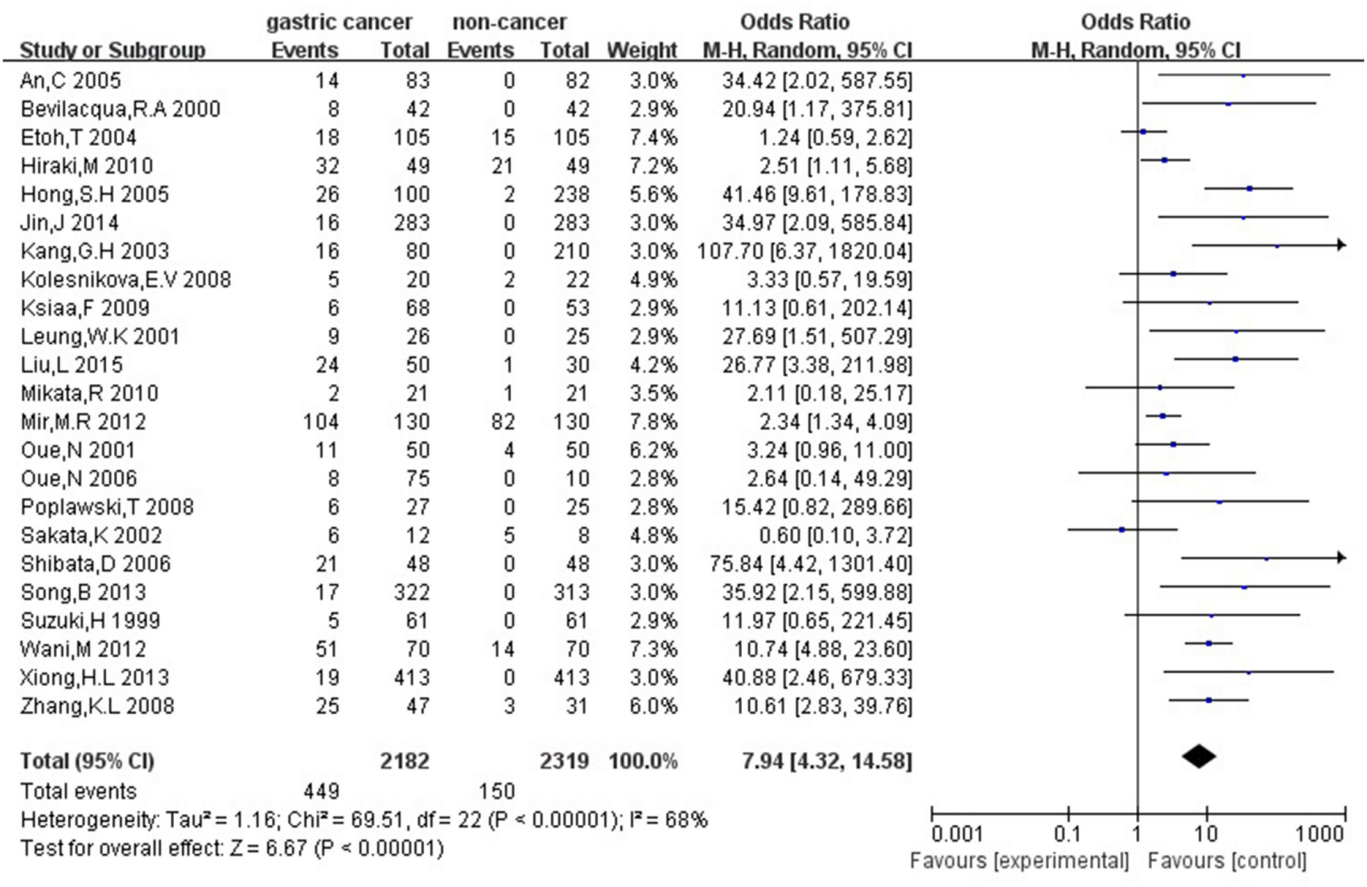
Forest plot concerning hMLH1 methylation and gastric cancer risk. The squares and horizontal lines represent the corresponding OR and 95% CI for each study. The area of the squares reflects the weight of each study. The diamond represents the pooled OR and 95% CI. Random-effect model was used.

Firstly, we analyzed the association between *hMLH1* promoter methylation and risk of gastric cancer. Significant heterogeneity of studies (*I*^2^ = 68%, *P* < 0.001) was detected by *X*^2^ test, so we employed random-effect model. In general, our result showed that the *hMLH1* methylation frequency in gastric cancer was obviously higher compared with non-cancer controls (OR = 7.94, 95%CI = 4.32–14.58, *P* < 0.001, Figure 2). We explored the sources of heterogeneity through subgroup analysis about the ethnicity, type of controls, specimen materials, and methods of detecting methylation (Table 2). There was no significant heterogeneity between different races (Asians subgroup and Caucasians subgroup), different specimen materials (tissue subgroup or other materials subgroup), or different methylation detection methods (MSP subgroup and other methods subgroup). However, remarkable heterogeneity between subgroup whose controls were autologous and subgroup whose controls were heterogeneous has been observed (*I*^2^ = 61.7%, *P* = 0.11).

**Table 2:** Stratified analysis of the association between hMLH1 methylation and gastric cancer risk.

**Groups**	**N**	**Methylation**		**Heterogeneity**		**Subgroup differences**	
		**OR (95%CI)**	**p**	**I^2^**	**P**	**I^2^**	**P**
Ethnicity						0%	0.86
Asian	15	7.82 (3.33–18.33)	<0.001	72%	<0.001		
Caucasians	8	8.87 (3.36–22.96)	<0.001	63%	0.008		
Control type						62%	0.11
Autologous	16	5.84 (2.96–11.53)	<0.001	67%	<0.001		
Heterogeneous	7	14.84 (6.00-36.70)	<0.001	31%	0.19		
Specimen materials						32%	0.22
Tissue	19	6.68 (3.50–12.74)	<0.001	67%	<0.001		
Others	4	16.00 (4.57–56.04)	<0.001	39%	0.18		
Method						0%	0.78
MSP	18	6.11 (4.61–8.09)	<0.001	72%	<0.001		
Others	5	6.75 (3.56–12.79)	<0.001	58%	0.05		

*N, the total number of eligible studies*.

**Figure 3:**
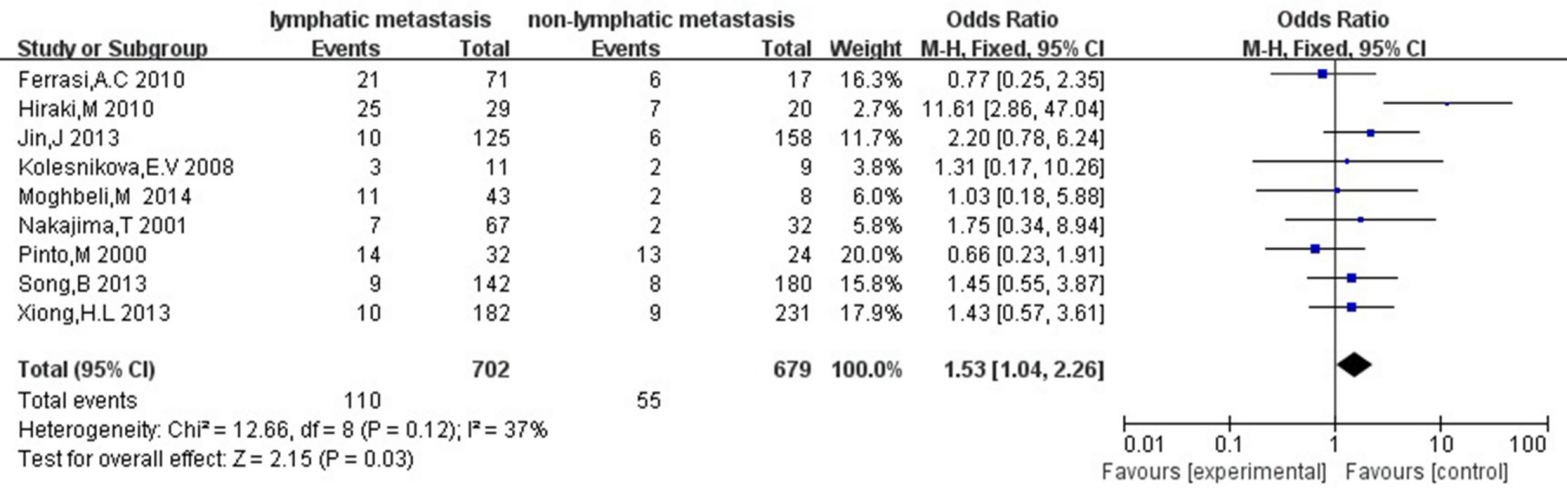
Forest plot concerning hMLH1 methylation and lymph node metastasis. Fixed-effect model was used.

**Figure 4:**
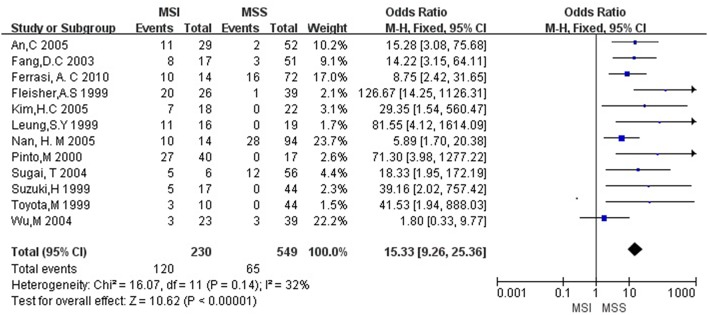
Forest plot concerning hMLH1 methylation and MSI. MSI, microsatellite instability. Fixed-effect model was used.

**Figure 5:**
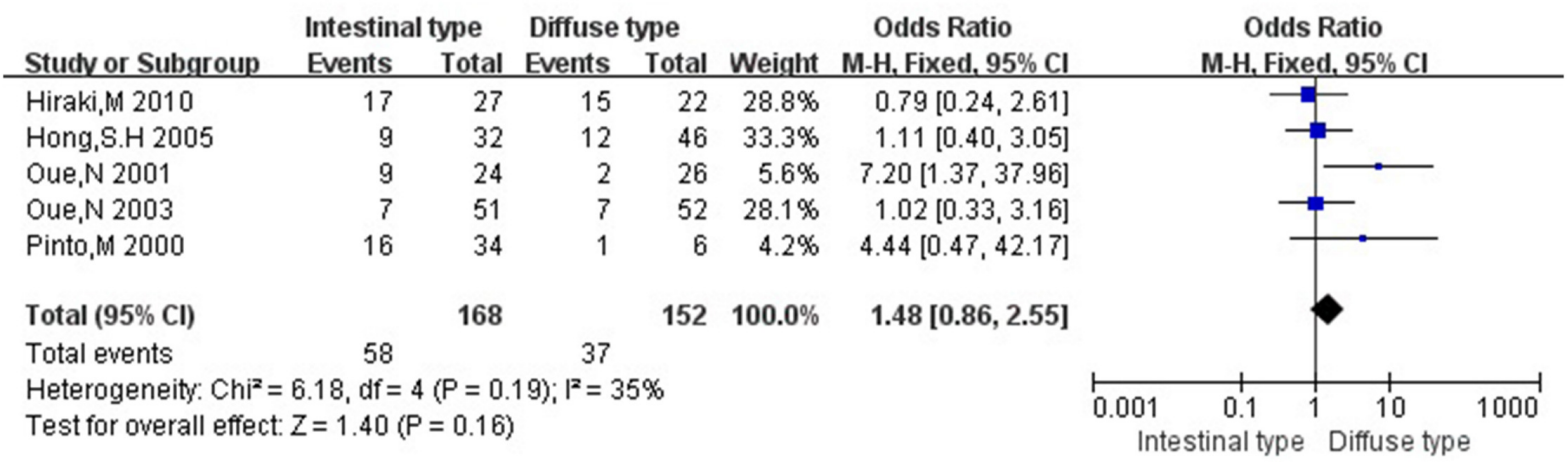
Forest plot concerning hMLH1 methylation and Lauren classification. Fixed-effect model was used.

**Figure 6:**
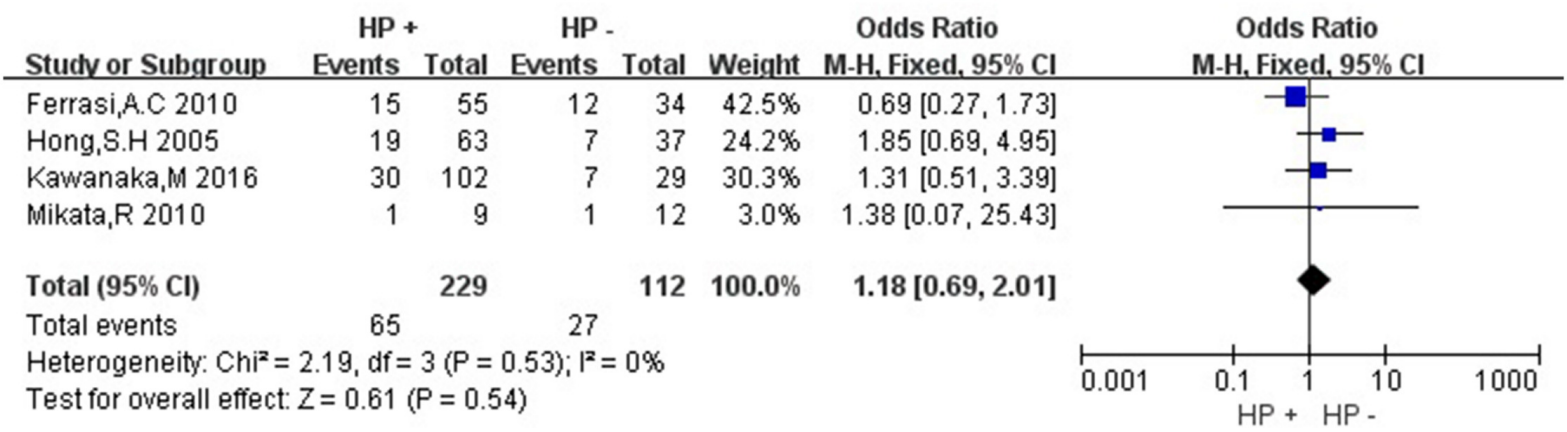
Forest plot concerning hMLH1 methylation and Helicobacter pylori infection. HP, Helicobacter pylori. Fixed-effect model was used.

**Figure 7:**
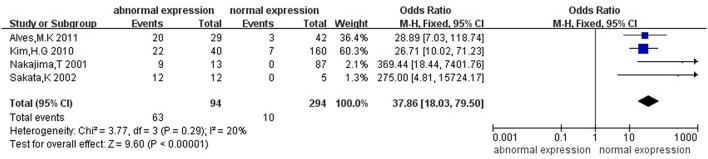
Forest plot concerning hMLH1 methylation and hMLH1 protein expression. Fixed-effect model was used.

Then we explored the associations between *hMLH1* promoter methylation and clinicopathological characteristics of gastric cancer. In short, there were significant statistical associations between *hMLH1* methylation and lymph node metastasis (OR = 1.53, 95%CI = 1.04–2.26, *P* = 0.03, fixed-effect model), microsatellite status (OR = 15.33, 95%CI = 9.26–25.36, *P* < 0.001, fixed-effect model) and low hMLH1 protein expression (OR = 37.86, 95%CI = 18.03–79.50, *P* < 0.001, fixed-effect model) in gastric cancer patients, but not with Lauren classification (OR = 1.48, 95%CI = 0.86–2.55, *P* = 0.16, fixed-effect model) or HP infection status (OR = 1.18, 95%CI = 0.69-2.01, *P* = 0.54, fixed-effect model). The results were listed in Table 3 and the forest plots were shown in Figures 3–7.

**Table 3:** The association between hMLH1 promoter methylation and clinicopathological characteristics of gastric cancer.

**Clinicopathologic characteristics**	**N**	**Cases**	**OR(95%CI)**	**P**	**I^2^ (%)**	**P_H_**
Lymph node metastasis	9	1381	1.53 (1.04–2.26)	0.03	37	0.12
Lauren classification	5	320	1.48 (0.86–2.55)	0.16	35	0.19
Microsatellite status	12	779	15.33 (9.26–25.36)	<0.001	32	0.14
HP infection	4	341	1.18 (0.69–2.01)	0.54	0	0.53
hMLH1 protein expression	4	388	37.86 (18.03–79.50)	<0.001	20	0.29

*N, the total number of eligible studies; P_H_, the p-value of Q test for heterogeneity among studies*.

### Sensitivity analysis and publication bias

**Figure 8:**
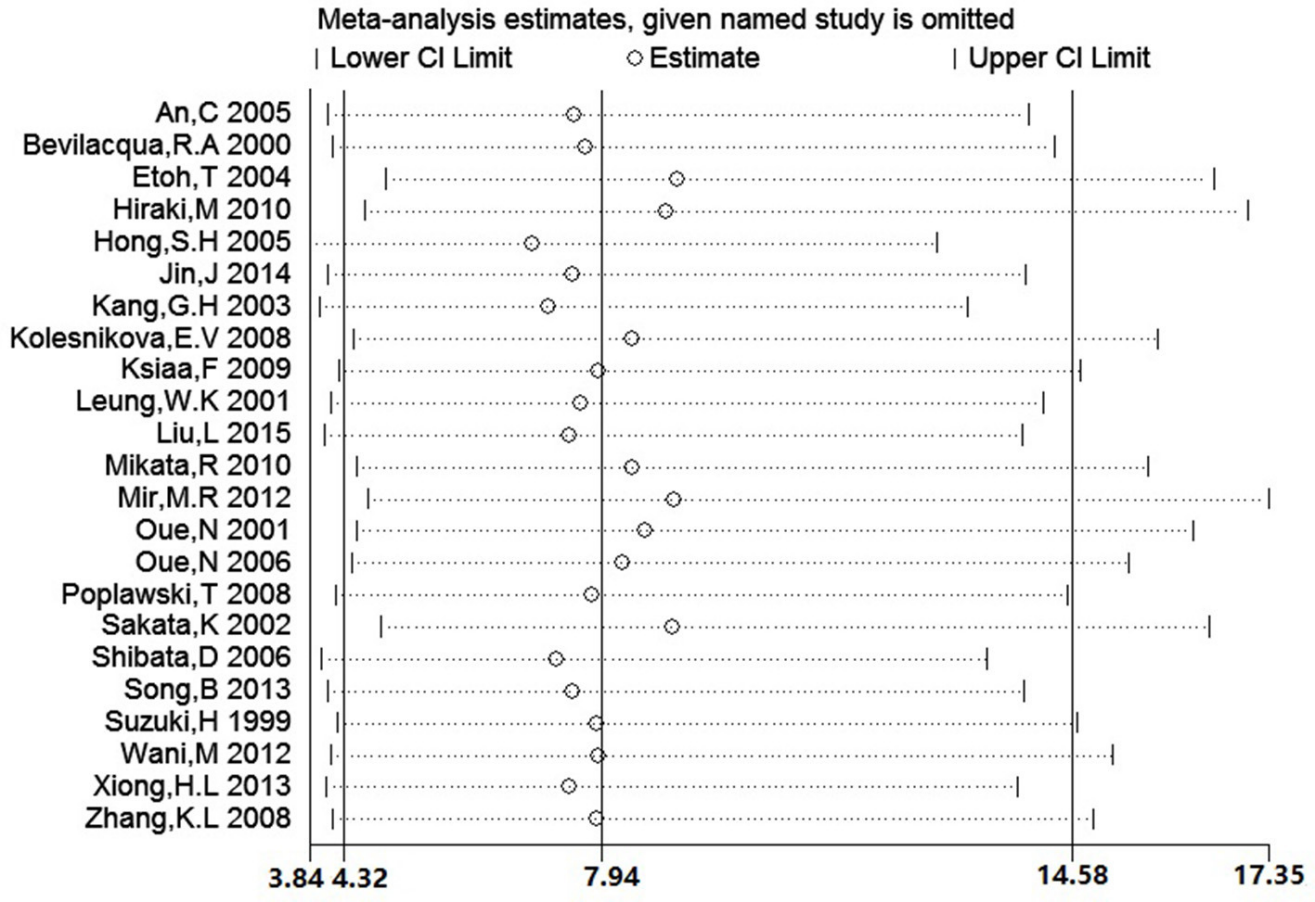
The plot of sensitivity analysis for evaluating the association between hMLH1 methylation and gastric risk. The circle and horizontal dashed line represent the pooled OR and 95% CI after omitting the corresponding study.

**Figure 9:**
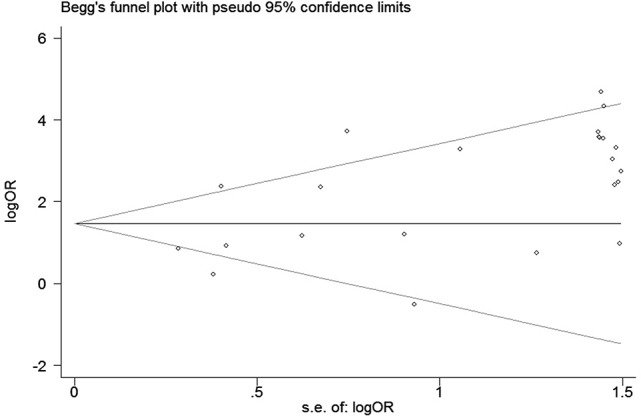
Funnel plot for evaluating the association of hMLH1 methylation with gastric cancer risk. Each circle represents one specific study.

We conducted a sensitivity analysis to evaluate the stability of our result by sequentially omitting every study from pooled analysis. The sensitivity analysis confirmed that the result was stable since omission of each single study could not significantly alter the pooled OR (Figure 8). We applied Begg's funnel plot to assess potential publication bias of these eligible articles. The shape of funnel plot was symmetric and *P* = 0.561, which indicated that no obvious publication bias was found. The funnel plot for evaluating the association of *hMLH1* promoter methylation with stomach cancer risk was shown in Figure 9.

## Discussion

Mismatch repairing is essential to ensure DNA replication fidelity, and mismatch repair deficiency will increase the chances of DNA mutation, which is important to the development of a tumor from normal cells (Thomas et al., 1996). Mismatch repair genes are a group of highly conserved housekeeping genes playing a major role in the maintenance of genetic and epigenetic stability (Li, 2008). *hMLH1* gene is one of the most widely studied. In recent years, some studies on cell lines suggested that methylation of *hMLH1* gene promoter is directly related to lack of hMLH1 protein expression, which is an important event in initiation of many kinds of sporadic tumors (Kane et al., 1997; Yao et al., 2004).

Previous studies have investigated the association between aberrant *hMLH1* promoter methylation and gastric cancer, yet the results were not consistent probably due to different ethnic groups, types of control, methylation detection methods and specimen materials. In order to obtain consistent results, we performed a meta-analysis involving 2,182 cases of tumors and 2,319 controls. The results showed that aberrant methylation of *hMLH1* promoter in stomach cancer tissues or bloods was positively associated with gastric cancer risk. Moreover, the frequency of *hMLH1* methylation was higher in gastric cancer with lymph node metastasis, microsatellite instability and absence of hMLH1 protein expression, suggesting that *hMLH1* promoter methylation might play a critical role in gastric cancer initiation and progression.

We confirmed that the frequency of *hMLH1* promoter methylation in gastric cancer was 7.94-fold higher than that in control groups, meanwhile, the expression of hMLH1 protein substantially decreased in stomach cancer patients with *hMLH1* hypermethylation. Our conclusion was similar to some results reported in other types of carcinomas (Mitchell et al., 2002; Han et al., 2016). *hMLH1* promoter methylation often leads to transcriptional silencing accompanied by down-regulation of mRNA expression, resulting in decrease of hMLH1 protein expression and mismatch repair dysfunction which contribute to tumorigenesis. Because of the heterogeneity of the studies, we conducted a subgroup analysis to explore the sources of heterogeneity. We found that there was heterogeneity between the autologous controls group and the heterogeneous controls group. The pooled OR was higher when the heterogeneous samples from non-cancer patients were used as the control group. There may be two explanations: (1) Gastric cancer is a systemic disease, certain changes may occur in healthy tissues more or less. (2) Different sampling methods among the studies: some used adjacent normal tissues as their autologous controls while the others chose remote normal tissues. We also found that *hMLH1* methylation was more frequent in gastric cancer patients with lymph node metastasis, indicated that *hMLH1* methylation may be implicated in the invasion and metastasis of gastric cancer.

Our meta-analysis also demonstrated that aberrant methylation of *hMLH1* was closely related to microsatellite instability. MSI was first found in hereditary nonpolyposis colorectal cancer (HNPCC) (Aaltonen et al., 1993), while gastric cancer possessed the highest prevalence of MSI (Ottini et al., 1997; Keller et al., 1998). It comprises length mutations in tandem oligonucleotide repeats which was believed to be caused by the inability of the MMR protein to fix a DNA replication error (Lynch and de la Chapelle, 2003). Indeed, MSI can be a molecular hallmark of mismatch-repair-deficient-tumors and even serve as a tool for the classification of gastric cancer (Simpson et al., 2001). We didn't find associations between *hMLH1* methylation and HP infection or Lauren classification, upon which researchers had different views. Nevertheless, the outcomes might be due to small sample sizes and need to be confirmed by more studies with larger samples in the future.

The sensitivity analysis and publication bias analysis results demonstrated that this meta-analysis was stable and had no obvious publication bias. However, our meta-analysis might still have some limitations. First of all, there was significant heterogeneity among these studies which were used to analyze the prevalence of *hMLH1* promoter methylation in gastric cancers, but we could not provide a good solution about sources of heterogeneity; Also, there may be differential effects in *hMLH1* methylation among different races, but these eligible studies did not contain all races, more researches are also needed to determine whether our outcomes are in consistent with studies about other ethnicities; Thirdly, we were not able to evaluate the associations between *hMLH1* promoter methylation and other clinicopathological features because of insufficient data, prospective population-based studies are necessary for further research.

Above all, this is the first meta-analysis focused on the association between aberrant *hMLH1* promoter methylation and gastric cancer, which provides evidence that silencing of the *hMLH1* gene by promoter hypermethylation is a major causative event in the occurrence and development of human gastric cancer. Nevertheless, more efforts are still needed to be made before regarding *hMLH1* promoter methylation as a potential diagnostic or prognostic biomarker.

## Author contributions

The literature searching, data extraction, statistical analysis, and paper writing were conducted by PY and YS. AL reviewed the manuscript. All authors approved the final version of the manuscript.

### Conflict of interest statement

The authors declare that the research was conducted in the absence of any commercial or financial relationships that could be construed as a potential conflict of interest. The reviewer SM and handling Editor declared their shared affiliation.
